# Supplementary material for: Pathogenicity of Avibacterium paragallinarum Strains from Peru and the Selection of Candidate Strains for an Inactivated Vaccine
Source: Vaccines (Basel). 2022 Jun 29;10(7):1043. doi: 10.3390/vaccines10071043 (PMC9318190; doi:10.3390/vaccines10071043)
Supplement: Supplementary file 1 [file vaccines-10-01043-s001.zip › vaccines-1741676-Supplementary.pdf]

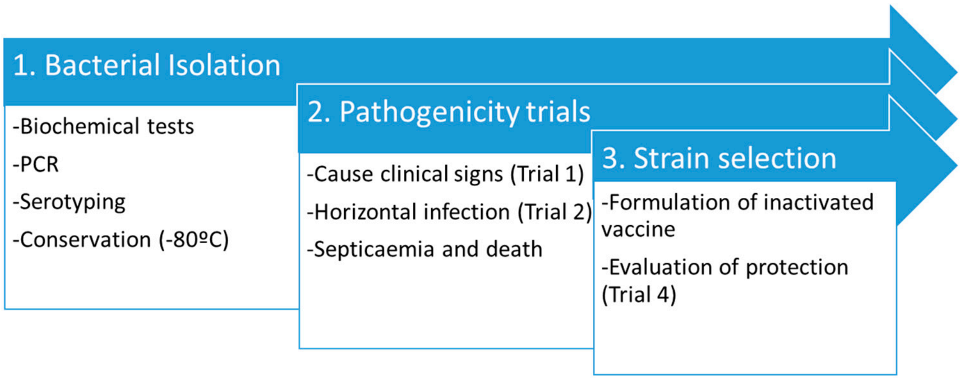

Figure S1. Experimental design. 1. *Avibacterium paragallinarum* strains were isolated from clinical cases of infectious coryza. These strains were identified by biochemical tests and PCR and kept frozen until usage. 2. Infection trials were performed to evaluate their capacity to cause clinical signs, horizontal infection as well as cause septicemia and death in experimentally inoculated chickens. 3. The most pathogenic strains were selected to formulate an inactivated vaccine that was tested for its capacity to protect chickens against experimental infection with *Avibacterium paragallinarum* of serogroups A, B, Bvar, and C.

Table S1 – List of *Avibacterium paragallinarum* strains isolated from infectious coryza outbreaks in Peru.

Strain N°	Province (City)	Chicken type	Year of isolation	Serogoup[1]
Q1	Lima	Breeders	1999	A
Q2	Lima	Laying hens	2006	A
Q3	Lima	Laying hens	2012	A
Q4	Lima	Laying hens	2008	A
Q5	Ica	Laying hens	2016	A
Q6	Lima	Broilers	2015	A
Q7	Lima	Broilers	2010	A/B[2]
Q8	Lima	Laying hens	2004	B
Q9	Loreto	Broilers	2005	B
Q10	Lima	Broilers	2006	B
Q11	Lima	Laying hens	2007	B
Q12	Lima	Broilers	2009	B
Q13	Lima	Broilers	2010	B
Q14	La Libertad	Laying hens	1998	B[3]
Q15	Ica	Laying hens	1999	B[3]
Q16	La Libertad	Laying hens	1999	B[3]
Q17	Arequipa	Broilers	1999	B[3]
Q18	San Martín	Broilers	2013	C
Q19	Ica	Breeders	2007	C
Q20	Lima	Broilers	2008	C
Q21	Lima	Broilers	2008	C
Q22	Lima	Laying hens	2009	C
Q23	La Libertad	Laying hens	2016	C[4]

Q24	Lima	Laying hens	2016	C

[1] All strains were serotyped according to the Page scheme [2], modified by Blackall et al. [4]
[2] Serotyping by inhibition of the haemagglutination test, using antisera of serogroups A and B, both of the international reference strains as well as the Argentinean strains could not discriminate between serogroup A or B.
[3] Variants of serogroup B [5].
[4] NAD independent.

Table S2 – Trial 1. Pathogenicity. Chickens were inoculated into the left infra-orbital sinus with 0.2 mL of each strain (n=20). Clinical signs score for all birds were observed on days 2 and 5 post-inoculation. Each bird was given between 0 and 4 points for each facial side. On day 5 post-inoculation, all birds were euthanized and both infra-orbital sinuses were cultured for the presence of *Avibacterium paragallinarum*. The total score for each strain is the sum of the clinical signs scores and the number of positive infra-orbital sinuses. No deaths were registered.

Strain	Inoculation Dose (CFU/mL)	Clinical signs scores* Days post-inoculation 2		5		Sum of Clinical Signs	Presence of *Av. paragallinarum**		Total score
		L	R	L	R		L	R	
Q1(A)	3x10^{7}	10	3	2	5	20	19	19	58
Q2(A)	2x10^{7}	32	15	0	3	50	20	18	88
Q3(A)	1x10^{8}	27	4	4	1	36	20	18	74
Q4(A)	6x10^{7}	2	0	1	0	3	20	20	43
Q5(A)	3x10^{7}	4	5	0	1	10	20	19	49
Q6(A)	8x10^{7}	2	0	0	0	2	20	20	42
Q7(A/B)	2x10^{7}	28	15	0	6	49	20	20	89
Q8(B)	1x10^{7}	34	4	14	1	53	20	20	93
Q9(B)	3x10^{7}	28	1	4	0	33	17	20	70
Q10(B)	2x10^{7}	39	3	11	6	59	18	19	96
Q11(B)	9x10^{6}	30	0	10	1	41	18	20	79
Q12(B)	1x10^{7}	35	0	7	1	43	20	20	83
Q13(B)	7x10^{6}	45	11	8	5	69	20	20	109
Q14(Bvar)	2x10^{7}	46	10	5	0	61	19	20	100
Q15(Bvar)	1x10^{7}	38	14	12	3	67	20	20	107
Q16(Bvar)	1x10^{7}	41	15	5	0	61	19	20	100
Q17(Bvar)	5x10^{7}	42	12	8	6	68	20	20	108
Q18(C)	1x10^{8}	55	2	23	3	83	20	17	120
Q19(C)	4x10^{7}	21	1	9	5	36	20	20	76
Q20(C)	1x10^{8}	42	2	13	1	58	20	19	97
Q21(C)	1x10^{8}	67	3	20	6	96	18	18	132
Q22(C)	6x10^{7}	47	2	10	1	60	20	20	100
Q23(C)	9x10^{7}	26	3	2	0	31	20	19	70
Q24(C)	1x10^{7}	44	3	7	5	59	20	19	98

* Left (L) or Right (R) infraorbital sinus.
CFU – Colony Forming Units

Table S3 – Trial 2. Horizontal infection. Part 1 – Serogroups A and B. Chickens were inoculated into the left infra-orbital sinus with 0.2 mL of each strain (n=8) and were housed in the same isolator with another 12 birds that were not inoculated. Clinical signs score for all birds were observed on days 2, 5, and 7 post-inoculation. Each bird was given between 0 and 4 points for each facial side. On day 7 post-inoculation, all birds were euthanized and both infra-orbital sinuses were cultured for the presence of *Avibacterium paragallinarum*. The total score for each strain is the sum of the clinical signs scores and the number of positive infra-orbital sinuses.

Strain (Serogroup)/ Inoculation Dose (CFU/mL)	n=	Inoculation	Clinical Signs Score (Day post-inoculation) 2		5		7		Total	Presence of *Av. paragallinarum*		Total Score
			L	R	L	R	L	R		L	R	
Q1 (A)	8	Yes	17	0	3	3	5	2	30	8	8	59
1,6x10^{7}	12	No	0	0	9	7	7	6	29	10	10	
Q2 (A)	8	Yes	29	6	13	5	10	3	66	6	6	87
4,2x10^{7}	12	No	0	0	8	3	5	5	21	8	8	
Q3 (A)	8	Yes	27	0	11	2	9	1	50	8	8	75
5x10^{7}	12	No	0	0	13	4	5	3	25	8	8	
Q4 (A)	8	Yes	24	0	9	4	12	3	52	8	8	74
6,7x10^{7}	12	No	0	0	7	5	7	3	22	10	10	
Q5 (A)	8	Yes	16	0	7	5	4	1	33	6	6	53
1,6x10^{8}	12	No	2	1	6	5	5	1	20	11	10	
Q6 (A)	8	Yes	16	0	4	0	8	1	29	6	6	39
8x10^{8}	12	No	0	0	5	2	3	0	10	10	6	
Q7 (A/B)	8	Yes	25	0	7	1	6	0	39	6	6	68
5x10^{7}	12	No	0	0	15	4	8	2	29	10	9	
Q8 (B)	8	Yes	7	1	4	0	3	0	15	5	5	17
2,7x10^{8}	12	No	1	0	1	0	0	0	2	2	2	
Q9 (B)	8	Yes	12	1	6	2	3	1	25	6	6	36
4x10^{8}	12	No	0	0	3	1	5	2	11	7	7	
Q10 (B)	8	Yes	17	1	11	2	6	3	40	5	5	45
5x10^{8}	12	No	1	0	2	1	1	0	5	6	5	
Q11 (B)	8	Yes	13	4	5	1	0	2	25	5	5	41
1,2x10^{8}	12	No	2	2	2	4	4	2	16	7	6	
Q12 (B)	8	Yes	9	4	1	0	0	1	15	3	3	19
2,5x10^{8}	12	No	1	1	1	1	0	0	4	2	0	
Q13 (B)	8	Yes	26	9	9	4	3	0	51	8	8	59
2x10^{8}	12	No	2	0	2	0	3	1	8	9	9	

* Left (L) or Right (R) infraorbital sinus.
CFU – Colony Forming Units

Table S4 – Trial 2. Horizontal infection. Part 2 – Serogroups Bvar and C. Chickens were inoculated into the left infra-orbital sinus with 0.2 mL of each strain (n=8) and were housed in the same isolator with another 12 birds that were not inoculated. Clinical signs score for all birds were observed on days 2, 5, and 7 post-inoculation. Each bird was given between 0 and 4 points for each facial side. On day 7 post-inoculation, all birds were euthanized and both infra-orbital sinuses were cultured for the presence of *Avibacterium*

paragallinarum. The total score for each strain is the sum of the clinical signs scores and the number of positive infra-orbital sinuses.

Strain (Serogroup)/ Inoculation Dose (CFU/mL)	n=	Inoculation	Clinical Signs Score (Day post-inoculation) 2		5		7		Total	Presence of *Av. paragallinarum*		Total Score
			L	R	L	R	L	R		L	R	
Q14 (Bvar)	8	Yes	19	0	2	0	0	0	21	6	7	42
$1,9x10^{8}$	12	No	1	0	7	8	3	2	21	12	12	
Q15 (Bvar)	8	Yes	17	2	7	4	4	4	38	7	7	66
$2,4x10^{8}$	12	No	1	0	7	8	5	7	28	12	9	
Q16 (Bvar)	8	Yes	25	1	10	2	5	0	43	7	7	75
$4,2x10^{8}$	12	No	0	0	10	10	6	6	32	12	12	
Q17 (Bvar)	8	Yes	25	0	9	2	2	0	38	6	7	57
$1,4x10^{8}$	12	No	1	0	4	9	3	2	19	10	9	
Q18 (C)	8	Yes	16	0	6	0	6	0	28	8	8	29
$1,1x10^{8}$	12	No	0	1	0	0	0	0	1	10	10	
Q19 (C)	8	Yes	12	0	3	0	1	1	17	8	8	18
$1,2x10^{8}$	12	No	0	0	1	0	0	0	1	10	9	
Q20 (C)	8	Yes	8	0	3	0	2	0	13	3	3	13
$3,5x10^{8}$	12	No	0	0	0	0	0	0	0	6	5	
Q21 (C)	8	Yes	11	0	2	0	3	0	16	7	6	21
$1,1x10^{8}$	12	No	1	0	3	1	0	0	5	11	11	
Q22 (C)	8	Yes	7	0	3	1	0	0	11	5	5	11
$2x10^{8}$	12	No	0	0	0	0	0	0	0	5	5	
Q23 (C)	8	Yes	27	0	8	3	3	1	42	7	7	64
$2,2x10^{8}$	12	No	0	0	2	8	4	8	22	12	11	
Q24 (C)	8	Yes	13	0	7	0	3	0	23	6	6	23
$2,2x10^{8}$	12	No	0	0	0	0	0	0	0	3	3	

* Left (L) or Right (R) infraorbital sinus.
CFU – Colony Forming Units

Table S5 - Trial 3. Septicaemia and death. Chickens were inoculated intraperitoneally with 0.5 mL of each strain (n=20). Mortality was registered until day 3 post-inoculation when all surviving birds were euthanized. Livers from dead or euthanized birds were cultured for the presence of *Avibacterium paragallinarum*.

Serogroup	Strain	Dose CFU/mL	Dead Birds*	Positive Livers*
A	Q02	$1.7x10^{8}$	7	18^{a}
	Q03	$7.8x10^{7}$	2	8^{b}
	Q07	$3.5x10^{7}$	4	18^{a}
B	Q10	$5.9x10^{8}$	0	2
	Q13	$2.3x10^{8}$	0	3
Bvar	Q14	$2.3x10^{8}$	3^{ab}	6^{a}
	Q15	$1.2x10^{8}$	1^{b}	1^{b}
	Q16	$2.1x10^{8}$	1^{b}	7^{a}
	Q17	$2.8x10^{8}$	6^{a}	11^{a}
C	Q18	$2.1x10^{7}$	4^{ab}	18^{a}

Q21	6.3×10^7	5[a]	12[a]
Q24	7.5×10^7	0[b]	4[b]

* For each serogroup, rates in the same column with different superscripts are significantly different by the chi-square test ($P < 0.05$)
